# GABA_B_ Receptor Antagonist CGP46381 Inhibits Form-Deprivation Myopia Development in Guinea Pigs

**DOI:** 10.1155/2015/207312

**Published:** 2015-01-11

**Authors:** Zhen-Ying Cheng, Xu-Ping Wang, Katrina L. Schmid, Yu-Fei Han, Xu-Guang Han, Hong-Wei Tang, Xin Tang

**Affiliations:** ^1^Clinical College of Ophthalmology, Tianjin Medical University, Tianjin Eye Hospital, 4 Gansu Road, Heping District, Tianjin 300020, China; ^2^Department of Ophthalmology, Qilu Hospital, Shandong University, Jinan, Shandong 250012, China; ^3^The Key Laboratory of Cardiovascular Remodeling and Function Research, Chinese Ministry of Education and Chinese Ministry of Health, Qilu Hospital, Shandong University, Jinan, Shandong 250012, China; ^4^School of Optometry and Vision Science, Faculty of Health, and Institute of Health and Biomedical Innovation, Queensland University of Technology, Brisbane, QLD 4059, Australia; ^5^Tianjin Nankai High School, 22 Nankaisima Road, Nankai District, Tianjin 300100, China; ^6^Department of Ophthalmology, The Second People's Hospital of Jinan, Jinan, Shandong 250001, China; ^7^Department of Ophthalmology, Liaocheng People's Hospital, Liaocheng, Shandong 252000, China

## Abstract

The aim was to investigate the effects of the GABA_B_ receptor antagonist, CGP46381, on form-deprivation myopia (FDM) in guinea pigs. Twenty-four guinea pigs had monocular visual deprivation induced using a diffuser for 11 days (day 14 to 25). The deprived eyes were treated with daily subconjunctival injections (100 *μ*l) of either 2% CGP46381, 0.2% CGP46381, or saline or received no injection. The fellow eyes were left untreated. Another six animals received no treatment. At the start and end of the treatment period, ocular refractions were measured using retinoscopy and vitreous chamber depth (VCD) and axial length (AL) using A-scan ultrasound. All of the deprived eyes developed relative myopia (treated versus untreated eyes, *P* < 0.05). The amount of myopia was significantly affected by the drug treatment (one-way ANOVA, *P* < 0.0001). The highest dose tested, 2% CGP46381, significantly inhibited myopia development compared to saline (2% CGP46381: −1.08 ± 0.40 D, saline: −4.33 ± 0.67 D, *P* < 0.01). The majority of these effects were due to less AL (2% CGP46381: 0.03 ± 0.01 mm, saline: 0.13 ± 0.02 mm, *P* < 0.01) and VCD (2% CGP46381: 0.02 ± 0.01 mm, saline: 0.08 ± 0.01 mm, *P* < 0.01) elongation. The lower dose tested, 0.2% CGP46381, did not significantly inhibit FDM (*P* > 0.05). Subconjunctival injections of CGP46381 inhibit FDM development in guinea pigs in a dose-dependent manner.

## 1. Introduction

Gamma-aminobutyric acid (GABA) is a major inhibitory neurotransmitter within the eye and brain [1, 2]. There are two main classes of GABA receptors: GABA_A_ receptors are ligand-gated ion channels (ionotropic receptors), whereas GABA_B_ receptors are G protein-coupled receptors (metabotropic receptors) [3, 4]. GABA_B_ receptors are comprised of two principal heterodimeric subunits, GABA_B1_ and GABA_B2_. GABA_B_ receptors via Gi/o proteins interact with neuronal inwardly rectifying potassium and voltage-gated calcium channels and when activated mediate slow synaptic inhibition [5].

In eyes, GABA_B_ receptors have been identified within the retina on photoreceptors, bipolar cells, amacrine cells, and ganglion cells [1, 6] and recently detected in chick retinal pigment epithelium [7]. In the retina, GABA_B_ receptors have been shown to modulate calcium currents in isolated goldfish retinal ganglion cells [8], modify acetylcholine and glycine release from amacrine cells in the rabbit retina [9], control arteriolar diameter in rat retinal whole-mounts [10], regulate chick retinal calcium waves during retinal development [11], and modify form-deprivation myopia (FDM) in chick eyes [12]. Although usually considered an inhibitory transmitter, the GABA_B_ receptor agonist baclofen facilitates the L-type calcium channel while inhibiting the N-type calcium current in isolated spiking retinal neurons from salamander retina [13].

CGP46381 is a water soluble GABA_B_ receptor antagonist of 219.26 molecular weight, IC_50_ of 4.9 *μ*M [14]. GABA_B_ antagonists alter neuronal brain activity; for example, CGP 36742 and CGP 51176 exhibit antidepression like effects in the forced swim test in mice [15] and SGS742 (CGP 36742) facilitates memory and cognition [16] in rats. The GABA_B_ antagonist CGP46381 is reported to antagonize the diminishing response to repeated auditory stimuli in rat hippocampus [17], inhibit suppression of hippocampal long-term potentiation and impair spatial learning in rats [18], stimulate spontaneous locomotor activity in mice [19], modify the contrast of the sensory input map in the olfactory receptor neuron terminals in mice [20], show proconvulsant activity of cortical epileptic after discharges in developing rat brain [21], and suppress absence seizures in the lethargic mutant mouse and rat models [22, 23].

CGP46381 has been shown to inhibit FDM in the chick model [12]. Other GABA antagonists shown to alter eye growth in chick include GABA_A_ (SR95531 [12], bicuculline [24]) and GABA_AOr_ (cis- and trans-3-ACPBPA [25] and TPMPA [12, 24, 26]) receptor antagonists. Additional GABA_B_ receptor antagonists shown to inhibit myopia in chick include SCH50911 and 2OH-saclofen [12]. Among all the GABA agents shown to inhibit myopia in chicks, cis- and trans-3-ACPBPA, TPMPA, and CGP46381 are the most effective [12, 25, 26]. Guinea pigs are a very useful mammalian eye growth model [27–30]. The GABA_AOr_ receptor antagonist TPMPA has been reported to inhibit FDM in guinea pigs [31, 32]. The aim was to determine whether CGP46381 (GABA_B_ antagonist) inhibits FDM in guinea pigs.

## 2. Methods

### 2.1. Treatments: FDM and Subconjunctival Injections

Thirty 14-day-old pigmented guinea pigs (Cavia porcellus) were obtained from Beijing Keyu Animal Centre (Beijing, China). Animals were reared under a 12-hour light/12-hour dark cycle (the light level was 1000 lux at the cage floor) in the animal facility. All guinea pigs had free access to food and water, and fresh cabbage was provided twice daily. Treatment and care of animals were conducted according to the ARVO Statement for the Use of Animals in Ophthalmic and Vision Research.

Twenty-four guinea pigs had monocular visual deprivation induced using a diffuser (random application, using velcro and tape, to the right or left eye) for 11 days (day 14 to 25) as previously described [29, 30, 33]. The deprived eyes were treated with daily subconjunctival injections (100 *μ*L) of either 2% CGP46381, 0.2% CGP46381, or saline or received no injection (*n* = 6). The fellow eye was left untreated. Another six animals received no treatment to either eye.

The concentration of CGP46381 was based on published work involving the chick myopia model and the physicochemical characteristics of CGP46381. Stone and coauthors [12] reported that intravitreal injection of CGP46381, at doses ranging from 1 *μ*g to 200 *μ*g, significantly inhibited the development of FDM and the associated vitreous chamber and axial elongation in chicks; the maximal antimyopia effect occurred at doses above 100 *μ*g. Subconjunctival application requires the injected agent to penetrate the sclera if it is to have intraocular effects; Dong and coauthors [28] calculated that only 1/10^4^ of the applied dose reached the inside of the guinea pig eye. The maximum water solubility of CGP46381 is 100 mM (equivalent to 2.2%); to avoid solubility issues 2% was chosen as the maximum concentration. Thus concentrations of 0.2% (100 *μ*L contains 200 *μ*g) and 2% were used.

CGP46381 (Tocris, Glasgow, UK) was dissolved in injectable saline and stored at −80°C until required. Subconjunctival injections were performed as previously described [28] after removal of the diffuser. The injection was performed under anaesthesia (2% isoflurane in oxygen; 26-gauge needle) once daily (9–11 AM) in a timely manner (less than 2 min) for 11 days. The injection site was the peripheral bulbar conjunctiva. After the injection, a drug bolus was observed under the conjunctiva. The diffuser was replaced immediately following the injection.

### 2.2. Measurements: Refraction, Vitreous Chamber Depth, and Axial Length

At the start and the end of the treatment refraction and eye length measurements were made. Refractive errors were measured using streak retinoscopy in awake animals following cycloplegia (3 drops of 1% cyclopentolate hydrochloride; Alcon, Fort Worth, TX) as previously described [28, 30]. Three readings were made along the horizontal and vertical meridians and the average of these, the spherical equivalent refraction (SER), used in subsequent data analyses.

Axial length (AL) and vitreous chamber depth (VCD) were measured using an A-scan ultrasonography (Suoer, 10 MHz; Tianjin, China) under anaesthesia (2% isoflurane in oxygen). The assumed conduction velocities were 1540 m/s [30]. The AL was defined as the distance between the front of the cornea and the inner limiting membrane of the retina. VCD was defined as the distance from the back of the lens to the inner retinal surface. Three ultrasound measurements comprising independent alignments of the probe were averaged and used in data analysis.

### 2.3. Data Analysis

Data were analyzed using the GraphPad Prism 5 (Version 5.01, GraphPad Software, Inc., USA). SER, VCD, and AL of treated and untreated eyes of guinea pigs within the same treatment group were analyzed using paired *t*-tests. The interocular differences in SER, AL, and VCD of eyes of guinea pigs in different treatment groups were analyzed using one-way ANOVA and Dunnett's post hoc test. Both the within group and between group differences were defined as significant at *P* < 0.05.

## 3. Results

At commencement of the experiment there were no significant differences in SER, AL, or VCD of treated and untreated eyes in each group (*P* > 0.05, paired *t*-test). There were also no significant differences in SER, AL, or VCD of eyes across different treatment groups (*P* > 0.05, one-way ANOVA). Similarly at the end of the experiment, there were no significant differences in SER, AL, or VCD of untreated eyes across the groups (*P* > 0.05, one-way ANOVA).

**Figure 1:**
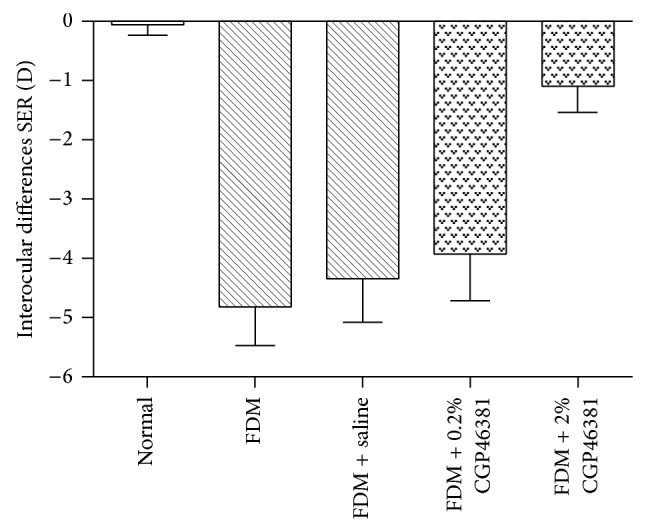
Effect of CGP46381 on spherical equivalent refraction (SER). Interocular differences (mean ± SD) in SER varied significantly with treatment (*P* < 0.0001). Compared to saline, 2% CGP46381 significantly inhibited the myopia (FDM + 2% CGP46381 versus FDM + saline, *P* < 0.01). The lower dose of CGP46381 0.2% was not effective at inhibiting myopia (*P* > 0.05).

At the end of the treatment period form-deprived eyes had developed relative myopia (*P* < 0.05, paired *t*-test). The interocular differences (treated minus untreated eye data) in SER were −0.04 ± 0.17 D in the normal group (neither eye treated), −4.79 ± 0.60 D in the FDM with no injection group, −4.33 ± 0.67 D in the FDM + saline group, −3.92 ± 0.72 D in the FDM + 0.2% CGP46381 group, and −1.08 ± 0.40 D in the FDM + 2% CGP46381 group (Figure 1). The amount of myopia induced was significantly affected by the drug treatment (one-way ANOVA, *F* = 74.61, *P* < 0.0001). Myopia was significantly less in the FDM + 2% CGP46381 group compared to that in FDM + saline group (Dunnett's post hoc, *P* < 0.01). There was no significant difference in relative myopia between the FDM + 0.2% CGP46381 group and the FDM + saline group (Dunnett's post hoc, *P* > 0.05). These data show that 2% CGP46381 subconjunctival injections inhibit FDM.

**Figure 2:**
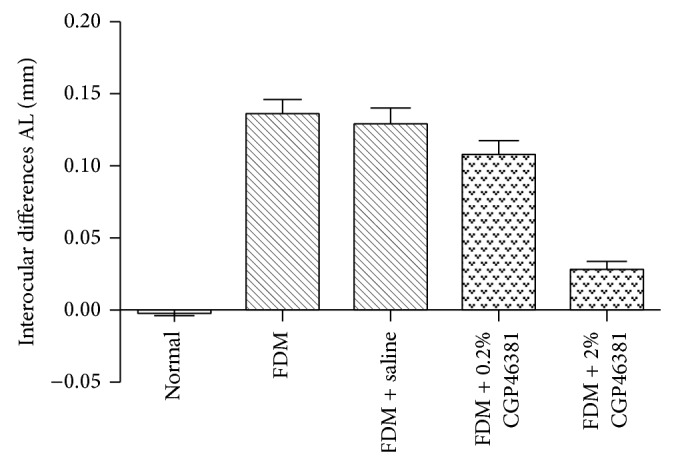
Effect of CGP46381 on axial length (AL). Interocular differences (mean ± SD) in AL varied significantly with treatment (*P* < 0.0001). Compared to saline, 2% CGP46381 significantly inhibited the AL elongation (FDM + 2% CGP46381 versus FDM + saline, *P* < 0.01). The lower dose of CGP46381 0.2% was not effective at inhibiting AL elongation (*P* > 0.05).

At the end of the treatment period the interocular differences in AL were −0.00 ± 0.01 mm in the normal group, 0.14 ± 0.02 mm in the FDM with no injection group, 0.13 ± 0.02 mm in the FDM + saline group, 0.11 ± 0.02 mm in the FDM + 0.2% CGP46381 group, and 0.03 ± 0.01 mm in the FDM + 2% CGP46381 group (Figure 2). The amount of AL elongation was significantly affected by the drug treatment (one-way ANOVA, *F* = 58.38, *P* < 0.0001). The amount of AL elongation of the form-deprived eyes in the FDM + 2% CGP46381 group was significantly less than that in FDM + saline group (Dunnett's post hoc, *P* < 0.01). There was no significant difference in AL elongation of form-deprived eyes of the FDM + 0.2% CGP46381 and FDM + saline groups (Dunnett's post hoc: 0.2% CGP46381 versus saline, *P* > 0.05).

**Figure 3:**
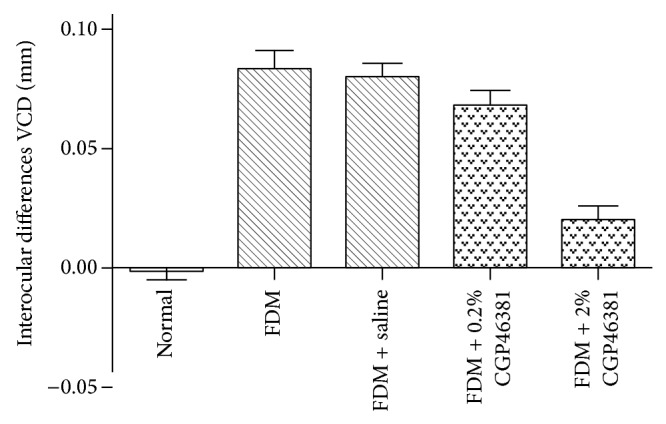
Effect of CGP46381 on vitreous chamber depth (VCD). Interocular differences (mean ± SD) in VCD varied significantly with treatment (*P* < 0.0001). Compared to saline, 2% CGP46381 significantly inhibited the VCD elongation (FDM + 2% CGP46381 versus FDM + saline, *P* < 0.01). The lower dose of CGP46381 0.2% was not effective at inhibiting the VCD elongation (*P* > 0.05).

At the end of treatment the interocular differences in VCD were −0.00 ± 0.01 mm in the normal group, 0.08 ± 0.02 mm in the FDM with no injection group, 0.08 ± 0.01 mm in the FDM + saline group, 0.07 ± 0.01 mm in the FDM + 0.2% CGP46381 group, and 0.02 ± 0.01 mm in FDM + 2% CGP46381 group (Figure 3). The amount of VCD elongation was significantly affected by the drug treatment (one-way ANOVA, *F* = 43.50, *P* < 0.0001). The VCD elongation in form-deprivation eyes in the FDM + 2% CGP46381 group was significantly reduced compared to the FDM + saline group (one-way ANOVA and Dunnett's test: 2% CGP46381 versus saline, *P* < 0.01). There was no significant difference in VCD elongation in form-deprivation eyes of the FDM + 0.2% CGP46381 group and the FDM + saline group (one-way ANOVA and Dunnett's test: 0.2% CGP46381 versus saline, *P* > 0.05). These data show that 2% CGP46381 subconjunctival injections inhibit the VCD elongation in FDM (Figure 3).

## 4. Discussion

We found that CGP46381, a GABA_B_ receptor antagonist, which inhibits experimental myopia development in chick models [12], also inhibits FDM in guinea pigs. Subconjunctival injections of 2% CGP46381 significantly inhibited the myopic shift and the elongation of AL and VCD. These data show that CGP46381 not only inhibits FDM in an avian model but also inhibits FDM in a mammalian (guinea pig) model.

Stone and coauthors [12] reported that intravitreal injection of CGP46381 inhibited FDM in chicks; the minimum effective dose was 1 *μ*g per day. Here we found that subconjunctival injection of CGP46381 inhibits FDM in guinea pigs; the effective dose was 100 *μ*L of 2% CGP46381 (2 mg per day), whereas the lower dose, 100 *μ*L of 0.2% CGP46381 (200 *μ*g per day), did not inhibit myopia. These data show that the effective antimyopia dose of CGP46381 applied via subconjunctival injection in guinea pigs is approximately 2000 times higher than that for intravitreal injection in chicks. These data are consistent with only 1/10^4^ of the applied subconjunctival dose reaching the vitreous chamber [26]. Leech and coauthors report that daily subconjunctival injections of pirenzepine were significantly less effective at inhibiting myopia than were intravitreal injections [34]. The concentration of CGP46381 found here to inhibit myopia can now be used in future investigations utilizing subconjunctival injections.

We previously reported that the GABA_AOr_ receptor antagonist TPMPA inhibits FDM in guinea pigs [32] and here we report that the GABA_B_ receptor antagonist CGP46381 has similar antimyopia effects. The maximal doses tested in both cases were similarly effective: 2% CGP46381 (91 mM) inhibited ~70% of the FDM and 1% TPMPA (62 mM) inhibited ~80% (*P* = 0.493). The effects of 2% CGP46381 and 1% TPMPA on the AL and VCD elongation were also similar (*P* = 0.200 and *P* = 0.243, resp.). The lower dose tested, 0.2% CGP46381 (9 mM), did not inhibit FDM whereas 0.3% TPMPA (19 mM) inhibited ~30%. The 0.3% dose of TPMPA was more effective than 0.2% CGP46381 at inhibiting myopia (*P* = 0.047) and VCD elongation (*P* = 0.012), while the degree of AL elongation was similar (*P* = 0.066). Stone and coauthors [12] reported that the minimum dose for TPMPA inhibiting FDM in chicks is 0.1 *μ*g and 1 *μ*g for CGP46381, and when a higher dose was used (e.g., 100 ug), the antimyopia effects for TPMPA and CGP46381 were similar. Our results in guinea pigs show correspondence to the results in chicks.

In this study, we found that CGP46381 inhibits FDM in guinea pigs, but the potential targets and possible mechanisms are not clear. GABA_B_ receptors have been detected in the retina [1] and RPE [7], which suggested that the retina and RPE would be the potential targets for CGP46381 inhibiting myopia. Sclera [35], choroid [36], RPE [37], and retina [38] are all implicated in myopia development, and the sclera determines the ocular size. How CGP46381 contacts with the GABA_B_ receptors in the retina and RPE and how the signals are then translated to the sclera to inhibit the eye elongation and myopia development require further investigation.

Several other kinds of neurotransmitter agents inhibit experimental myopia in a variety of animal models, including the muscarinic acetylcholine receptor nonspecific antagonist atropine (in chicks [39], mice [40], and monkeys [41]), the M1 receptor antagonist pirenzepine (in chicks [34], guinea pigs [27], and monkeys [41]), the M4 muscarinic antagonist MT-3 (in chicks [42] and tree shrews [43]), the dopamine receptor agonist, and apomorphine (in chicks [33, 44], guinea pigs [28], and monkeys [45]). Thus GABA must be involved in a complicated eye growth pathway involving many retinal cells and associated transmitters.

In summary, we found that subconjunctival injection of the GABA_B_ receptor antagonist CGP46381 can effectively inhibit FDM in guinea pigs. Whether CGP46381 inhibits myopia in the other animal models and humans requires further investigation. The potential targets and possible mechanisms via which CGP46381 inhibits myopia remain unexplored.

## Abbreviations

CGP46381: (3-Aminopropyl)(cyclohexylmethyl)phosphinic acid
FDM: Form-deprivation myopia
GABA: Gamma-aminobutyric acid
AL: Axial length
VCD: Vitreous chamber depth.

## References

[1] Yang X.-L. (2004). Characterization of receptors for glutamate and GABA in retinal neurons. *Progress in Neurobiology*.

[2] Wang D. D., Kriegstein A. R. (2009). Defining the role of GABA in cortical development. *Journal of Physiology*.

[3] Chebib M., Johnston G. A. R. (1999). The ‘ABC’ of GABA receptors: a brief review. *Clinical and Experimental Pharmacology and Physiology*.

[4] Olsen R. W., Sieghart W. (2008). International Union of Pharmacology. LXX. Subtypes of gamma-aminobutyric acid(A) receptors: classification on the basis of subunit composition, pharmacology, and function. Update. *Pharmacological Reviews*.

[5] Marshall F. H., Jones K. A., Kaupmann K., Bettler B. (1999). GABA(B) receptors—the first 7TM heterodimers. *Trends in Pharmacological Sciences*.

[6] Slaughter M. M., Pan Z. H. (1992). The physiology of GABAB receptors in the vertebrate retina. *Progress in Brain Research*.

[7] Cheng Z.-Y., Wang X.-P., Schmid K. L., Liu L. (2013). Identification of GABA receptors in chick retinal pigment epithelium. *Neuroscience Letters*.

[8] Bindokas V. P., Ishida A. T. (1991). (-)-baclofen and *γ*-aminobutyric acid inhibit calcium currents in isolated retinal ganglion cells. *Proceedings of the National Academy of Sciences of the United States of America*.

[9] Neal M. J., Cunningham J. R. (1995). Baclofen enhancement of acetylcholine release from amacrine cells in the rabbit retina by reduction of glycinergic inhibition. *The Journal of Physiology*.

[10] Hinds K., Monaghan K. P., Frolund B. (2013). GABAergic control of arteriolar diameter in the rat retina. *Investigative Ophthalmology & Visual Science*.

[11] Catsicas M., Mobbs P. (2001). GABAB receptors regulate chick retinal calcium waves. *The Journal of Neuroscience*.

[12] Stone R. A., Liu J., Sugimoto R., Capehart C., Zhu X., Pendrak K. (2003). GABA, experimental myopia, and ocular growth in chick. *Investigative Ophthalmology & Visual Science*.

[13] Shen W., Slaughter M. M. (1999). Metabotropic GABA receptors facilitate L-type and inhibit N-type calcium channels in single salamander retinal neurons. *The Journal of Physiology*.

[14] Olpe H.-R., Steinmann M. W., Ferrat T. (1993). The actions of orally active GABA_B_ receptor antagonists on GABAergic transmission in vivo and in vitro. *European Journal of Pharmacology*.

[15] Nowak G., Partyka A., Pałucha A. (2006). Antidepressant-like activity of CGP 36742 and CGP 51176, selective GABA_B_ receptor antagonists, in rodents. *British Journal of Pharmacology*.

[16] Helm K. A., Haberman R. P., Dean S. L. (2005). GABAB receptor antagonist SGS742 improves spatial memory and reduces protein binding to the cAMP response element (CRE) in the hippocampus. *Neuropharmacology*.

[17] Hershman K. M., Freedman R., Bickford P. C. (1995). GABA_B_ antagonists diminish the inhibitory gating of auditory response in the rat hippocampus. *Neuroscience Letters*.

[18] Brucato F. H., Levin E. D., Mott D. D., Lewis D. V., Wilson W. A., Swartzelder H. S. (1996). Hippocampal long-term potentiation and spatial learning in the rat: effects of GABA_B_ receptor blockade. *Neuroscience*.

[19] Colombo G., Melis S., Brunetti G. (2001). GABA_B_ receptor inhibition causes locomotor stimulation in mice. *European Journal of Pharmacology*.

[20] Vučinić D., Cohen L. B., Kosmidis E. K. (2006). Interglomerular center-surround inhibition shapes odorant-evoked input to the mouse olfactory bulb in vivo. *Journal of Neurophysiology*.

[21] Mareš P. (2013). Proconvulsant action of two GABA_B_ receptor antagonists is age-dependent. *Physiological Research*.

[22] Hosford D. A., Wang Y., Snead O. C. (1995). Characterization of the antiabsence effects of SCH 50911, a GABA(B) receptor antagonist, in the lethargic mouse, *γ*-hydroxybutyrate, and pentylenetetrazole models. *Journal of Pharmacology and Experimental Therapeutics*.

[23] Aizawa M., Ito Y., Fukuda H. (1997). Pharmacological profiles of generalized absence seizures in lethargic, stargazer and *γ*-hydroxybutyrate-treated model mice. *Neuroscience Research*.

[24] Leung C. K. S., Yeung C.-K., Chiang S. W. Y., Chan K. P., Pang C. P., Lam D. S. C. (2005). GABAA and GABAC (GABAA0r) receptors affect ocular growth and form-deprivation myopia. *Cutaneous and Ocular Toxicology*.

[25] Chebib M., Hinton T., Schmid K. L. (2009). Novel, potent, and selective GABAC antagonists inhibit myopia development and facilitate learning and memory. *Journal of Pharmacology and Experimental Therapeutics*.

[26] Schmid K. L., Strasberg G., Rayner C. L., Hartfield P. J. (2013). The effects and interactions of GABAergic and dopaminergic agents in the prevention of form deprivation myopia by brief periods of normal vision. *Experimental Eye Research*.

[27] Le Q.-H., Cheng N.-N., Wu W., Chu R.-Y. (2005). Effects of pirenzepine ophthalmic solution on form-deprivation myopia in the guinea pigs. *Chinese Medical Journal*.

[28] Dong F., Zhi Z., Pan M. (2011). Inhibition of experimental myopia by a dopamine agonist: different effectiveness between form deprivation and hyperopic defocus in guinea pigs. *Molecular Vision*.

[29] Backhouse S., Phillips J. R. (2010). Effect of induced myopia on scleral myofibroblasts and in vivo ocular biomechanical compliance in the guinea pig. *Investigative Ophthalmology and Visual Science*.

[30] Howlett M. H., McFadden S. A. (2006). Form-deprivation myopia in the guinea pig (*Cavia porcellus*). *Vision Research*.

[31] McFadden S. A., Gambrill R., Leotta A. J. (2011). Inhibition of GABAc slows ocular growth and myopia in the mammalian eye. *Investigative Ophthalmology & Visual Science*.

[32] Cheng Z.-Y., Wang X.-P., Schmid K. L., Han X.-G. (2014). Inhibition of form-deprivation myopia by a GABAAOr receptor antagonist, (1,2,5,6-tetrahydropyridin-4-yl) methylphosphinic acid (TPMPA), in guinea pigs. *Graefe's Archive for Clinical and Experimental Ophthalmology*.

[33] Schmid K. L., Wildsoet C. F. (2004). Inhibitory effects of apomorphine and atropine and their combination on myopia in chicks. *Optometry and Vision Science*.

[34] Leech E. M., Cottriall C. L., McBrien N. A. (1995). Pirenzepine prevents form deprivation myopia in a dose dependent manner. *Ophthalmic and Physiological Optics*.

[35] McBrien N. A., Jobling A. I., Gentle A. (2009). Biomechanics of the sclera in myopia: extracellular and cellular factors. *Optometry and Vision Science*.

[36] Summers J. A. (2013). The choroid as a sclera growth regulator. *Experimental Eye Research*.

[37] Rymer J., Wildsoet C. F. (2005). The role of the retinal pigment epithelium in eye growth regulation and myopia: a review. *Visual Neuroscience*.

[38] Stone R. A., Pardue M. T., Iuvone P. M., Khurana T. S. (2013). Pharmacology of myopia and potential role for intrinsic retinal circadian rhythms. *Experimental Eye Research*.

[39] McBrien N. A., Moghaddam H. O., Reeder A. P. (1993). Atropine reduces experimental myopia and eye enlargement via a nonaccommodative mechanism. *Investigative Ophthalmology & Visual Science*.

[40] Barathi V. A., Beuerman R. W. (2011). Molecular mechanisms of muscarinic receptors in mouse scleral fibroblasts: prior to and after induction of experimental myopia with atropine treatment. *Molecular Vision*.

[41] Tigges M., Iuvone P. M., Fernandes A. (1999). Effects of muscarinic cholinergic receptor antagonists on postnatal eye growth of rhesus monkeys. *Optometry and Vision Science*.

[42] Mcbrien N. A., Arumugam B., Gentle A., Chow A., Sahebjada S. (2011). The M4 muscarinic antagonist MT-3 inhibits myopia in chick: evidence for site of action. *Ophthalmic and Physiological Optics*.

[43] Arumugam B., McBrien N. A. (2012). Muscarinic antagonist control of myopia: evidence for M4 and M1 receptor-based pathways in the inhibition of experimentally-induced axial myopia in the tree shrew. *Investigative Ophthalmology and Visual Science*.

[44] Rohrer B., Spira A. W., Stell W. K. (1993). Apomorphine blocks form-deprivation myopia in chickens by a dopamine D2-receptor mechanism acting in retina or pigmented epithelium. *Visual Neuroscience*.

[45] Iuvone P. M., Tigges M., Stone R. A., Lambert S., Laties A. M. (1991). Effects of apomorphine, a dopamine receptor agonist, on ocular refraction and axial elongation in a primate model of myopia. *Investigative Ophthalmology and Visual Science*.

